# 

**Published:** 1872-03-01

**Authors:** 


THE American Medical Association will hold its 23d Annual Session at Horticultural Hall, Broad St., Philadelphia, commencing at 11 o'clock A. M., May 7, 1872.—*Med. News.*

GENEVA MEDICAL COLLEGE.—The Commencement exercises of this college were held Jan. 23d, at Linden Hall. The degree of M. D. was conferred upon twelve candidates. With these exercises this institution ceases to exist.

A majority of the faculty have accepted chairs in the "College of Physicians and Surgeons of the Syracuse University." This institution will open for the reception of students some time during the current year.—*Med. News.*

